# Pediatric patient with a bilateral Salter-Harris II fracture and slipped capital femoral epiphysis secondary to autosomal recessive osteopetrosis

**DOI:** 10.1007/s00132-022-04278-x

**Published:** 2022-07-08

**Authors:** Ayham Jaber, Martin Schwarze, Verena Steinle, Marco Götze, Sébastien Hagmann

**Affiliations:** 1 Department of Orthopedics and Trauma Surgery, Center for Orthopedics, Trauma Surgery and Spinal Cord Injury, Heidelberg University Hospital, Schlierbacher Landstr. 200a, 69118 Heidelberg, Germany; 2 Department of Diagnostic and interventional Radiology, Heidelberg University Hospital, Im Neuenheimer Feld 420, 69120 Heidelberg, Germany

**Keywords:** Femoral neck fracture, Osteosynthesis, Congenital disease, Osteotomy, Pathologic fracture, Growth plate, Schenkelhalsfraktur, Osteosynthese, Kongenitale Erkrankung, Osteotomie, Pathologische Fraktur, Wachstumsfuge

## Abstract

Treatment of femoral neck fractures secondary to osteopetrosis is an uncertain and puzzled decision. Experience in the treatment, especially in the pediatric population, is scarcely reported. The duration of conservative treatment is prolonged and poses the risks of non-union and development of coxa vara deformity. The recommended treatment is closed reduction and internal fixation; however, surgery on osteopetrotic bone is challenging due to defective bone marrow function, delayed consolidation and higher risk of intraoperative fractures. Slipped capital femoral epiphysis secondary to osteopetrosis is very rarely reported. This article presents the case of a 5-year-old female patient with rapidly deteriorating physical function due to bilateral proximal femoral Salter-Harris type II fractures with associated slippage of the growth plates secondary to confirmed autosomal recessive osteopetrosis. Operative treatment was performed in a tertiary level orthopedic center with closed reduction and internal fixation with cannulated screws. A loss of fixation with coxa vara deformity was seen on the left side 7 months postoperatively with increasing pain. A revision surgery with reosteosynthesis and a valgus osteotomy was thus performed which showed good subjective and objective results 1 year postoperatively with complete bony union.

## Abstract (de)

Die Behandlung von Schenkelhalsfrakturen als Folge einer Osteopetrose ist eine Herausforderung. Erfahrungen mit der Behandlung, insbesondere bei Kindern und Jugendlichen, liegen kaum vor. Die Dauer der konservativen Therapie ist verlängert und birgt das Risiko einer Pseudarthrose und der Entwicklung einer Coxa-vara-Deformität. Die empfohlene Behandlung ist eine geschlossene Reposition und interne Fixation. Die osteopetrotischen Knochen sind jedoch aufgrund der defekten Knochenmarkfunktion, der verzögerten Konsolidierung und des höheren Risikos für intraoperative Frakturen eine Herausforderung. Sehr selten wird über eine Epiphyseolysis capitis femoris bei Osteopetrose berichtet. Dieser Fall stellt eine 5‑jährige Patientin mit rasch nachlassender körperlicher Funktionsfähigkeit aufgrund einer bilateralen Salter-Harris-II-Fraktur mit beidseitiger Epiphyseolysis capitis femoris als Folge einer bestätigten autosomal-rezessiven Osteopetrose vor. Die operative Behandlung wurde in einem Universitätsklinikum durchgeführt mit geschlossener Reposition und interner Fixation mit kannelierten Schrauben. Sieben Monate postoperativ zeigte sich auf der linken Seite ein Fixationsverlust mit Coxa vara und zunehmenden Schmerzen. Daher wurde eine Revisionsoperation mit Reosteosynthese und einer Valgisierungsosteotomie durchgeführt, die 1 Jahr postoperativ gute subjektive und objektive Ergebnisse mit einer vollständigen Knochenheilung zeigte.

## Introduction

OP is an old term that originates from the Greek words *osteo* (bone) and *petros* (stone). It was first introduced by Albers-Schoenberg in 1904 [1]. The oldest reported case of OP in a human skeleton dates back to 4620–4456 BCE [2]. This rare hereditary disease is also referred to as Albers-Schönberg disease or marble bone disease. It is characterized by altered bone resorption which leads to increased bone mass and presents as generalized osteosclerosis. The OP varies in severity and is classified into three main variants [3]. ADO is the benign and most common form with an incidence of 1 in 20,000 births [4]. Around 40% of patients with ADO remain asymptomatic during their lifetime [5]. Two less common but more fetal variants exist which include infantile malignant and intermediate forms. The infantile malignant form is inherited in an autosomal recessive inheritance pattern. It presents in the early childhood years with a low life expectancy due to bone marrow insufficiency and advanced disease complications [6]. The intermediate form can be inherited both as autosomal recessive as well as autosomal dominant pattern. It usually presents during the first decade of life. ARO has an incidence of 1 in 250,000 child births [7].

A pathological fracture is one of the most common manifestations of OP, accounting for around 75% of all presentations. Other usual skeletal presentations include back pain, bone pain, and deformities such as coxa vara or genu varum [8]. An OP can also cause a wide range of extraskeletal symptoms [9]. Osteopetrotic bone cannot adequately mobilize its calcium pool. This could lead to hypocalcemia and resultant tetany and seizures [10]. Dental abnormalities can also occur such as tooth agenesis, failure of tooth eruption and tooth loss due to early decay and caries [11]. Neuropathies result due to progressive constriction of the cranial foramina or failure of the foramina to enlarge proportionally with growth [12]. The optic nerve is most commonly affected in patients with ARO, resulting in optic nerve atrophy and visual impairment. The facial nerve is also commonly afflicted. Extrinsic zone compression could lead to sensorineural deafness in a manner similar to other cranial nerve palsies. Extramedullary hematopoiesis secondary to anemia could develop as a result of the embouchement of osteopetrotic bone on the bone cavity, resulting in hepatosplenomegaly. This eventually leads to bone marrow failure and death [13].

The diagnosis of OP can be done radiologically relying on characteristic findings such as in the present case. Genetic testing adds important information by identifying mutations associated with unique disease complications. OP is caused by mutations in at least 10 genes which have been identified as causative in humans, accounting for 70% of all cases. ADO is caused by a chloride channel 7 (*CLCN7*) chromosomal gene mutation [9]. The TCIRG1 gene encodes a subunit of a large protein complex known as a vacuolar H+-ATPase (V-ATPase). V‑ATPase-dependent organelle acidification is necessary for several intracellular processes. Mutations in this gene are associated with infantile malignant osteopetrosis [14]. Recent advances in hematopoietic stem cell transplantation (HSCT) have significantly improved life expectancy and quality of life of osteopetrotic patients, with the 10-year survival rate reaching as high as 62%, achieving acceptable social function [15]. Evidence of skeletal remodeling in patients with OP following HSCT has also been reported [16].

Several reports of femoral neck fractures secondary to OP exist in the medical literature, yet these are not sufficient to form conclusive guidelines regarding treatment. According to the Pediatric Orthopedic Society of North America, surgical treatment is the recommended approach [17]. However, the realization is difficult in the setting of OP. As a result, complications such as hardware failure, osteomyelitis, prosthetic fractures, and pseudarthrosis are common [18, 19]. Results of both conservative as well as surgical treatment were reported with mixed results [20, 21]. The medical literature lacks experience of surgical treatment in pediatric patients with femoral neck fractures [21–23]. Most reported cases are of adult patients [23]. Furthermore, only 2 cases in the literature reported a slipped capital femoral epiphysis (SCFE) in a pediatric patient with ADO [24, 25]. No such cases were reported in association with ARO. We present a case of a 5-year-old patient with Salter-Harris II fractures associated SCFE due to confirmed ARO treated surgically, revised 7 months postoperatively and followed up for 1 year following revision.

## Case

**Fig. 1:**
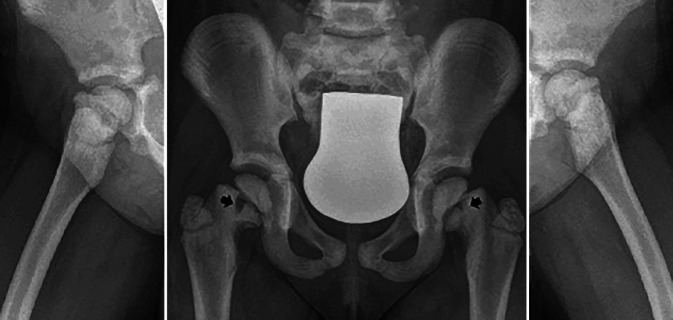
Preoperative anteroposterior view of the pelvis and Lowenstein radiograph showing bilateral femoral neck fractures (Black arrows) with grade I slippage of the growth plates

**Fig. 2:**
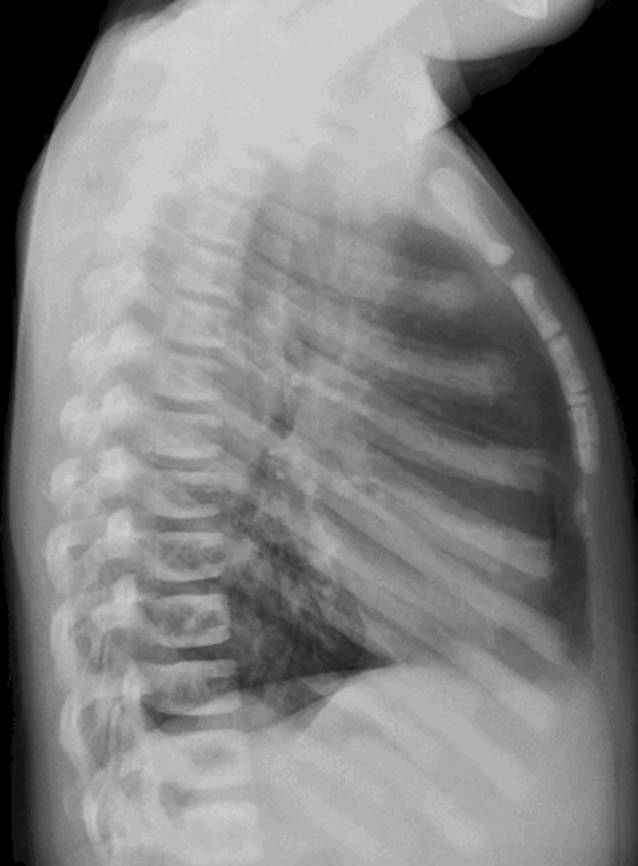
Lateral chest radiograph showing generalized increase in bone density with typical sclerosis of vertebral endplates resulting in “sandwich vertebrae” appearance and widening of the ribs at the costochondral junction

**Fig. 3:**
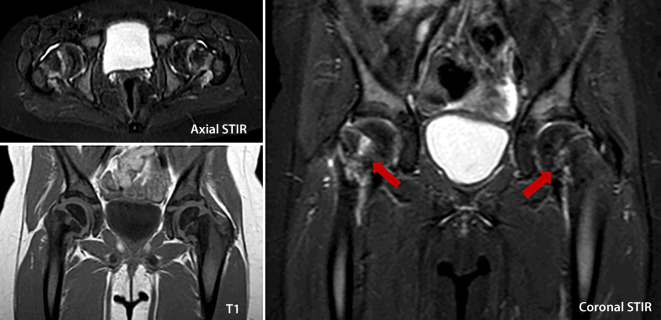
Preoperative MR imaging of the pelvis: STIR sequence axial and coronary shows clear bone edema in both femoral necks (Red arrows) with a T1 correlate, compatible with bilateral femoral neck fracture and slight hip joint effusion. *STIR* Short tau inversion recovery

A 5-year-old female patient was referred to our pediatric orthopedic department in July 2020 with a history of limping as well as pain radiating to both knees. The symptoms started mildly 2 months prior with gradual worsening. The initial visit to the primary physician did not include a radiological examination in order to spare radiation exposure for the young patient since no trauma was reported. Physical therapy was done but with no improvement. The patient eventually could not bear weight anymore and was mobilized with a wheelchair. Radiographs of both hips were then done and revealed bilateral medial pathologic Salter-Harris II fractures with minor slippage of both growth plates as shown in Fig. 1 (grade I according to Southwick) [26]. Moreover, generalized osteosclerotic bone with cortical thickening and medullary calcifications were evident. Radiography of the thorax demonstrated sclerosis of vertebral endplates resulting in a “sandwich vertebrae” appearance and widening of the ribs at the costochondral junction (Fig. 2). Magnetic resonance imaging (MRI) was performed to rule out other etiologies (Fig. 3). There was no family history of marble bones or other disorders of bone growth. Genetic examination showed novel mutations of the TCIRG1 gene in the patient and both parents, confirming the diagnosis of ARO [14].

**Fig. 4:**
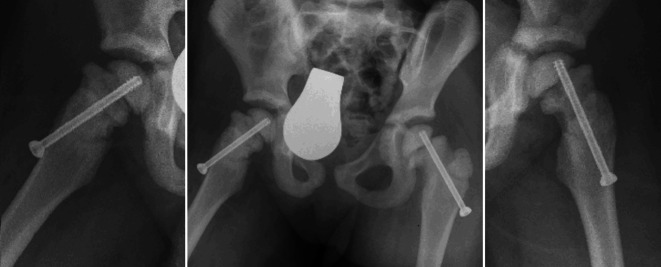
Postoperative anterior posterior and Lowenstein radiographs after osteosynthetic treatment of the bilateral femoral neck fractures

**Fig. 5:**
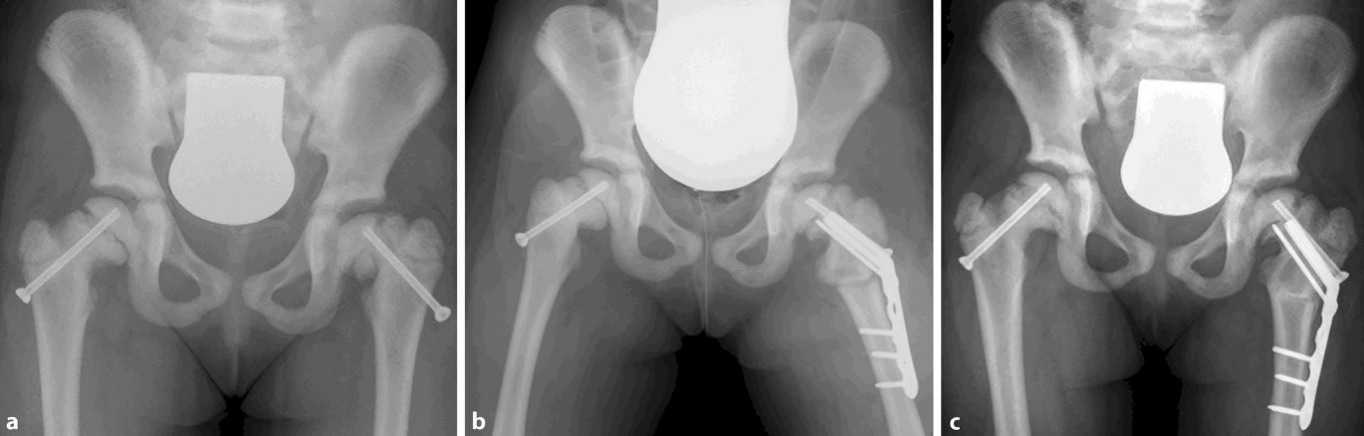
**a** Anteroposterior radiograph 7 months postoperatively showing a loss of fracture reposition on the left side with a coxa vara deformity and minor increased slippage of the epiphyseal plate. **b** Postoperative radiograph following revision surgery with re-osteosynthesis and a valgus osteotomy on the left side. **c** Radiograph 1 year postoperatively showing complete bony union of the osteotomy and no loss of correction at the femoral neck

Various treatment options were proposed to the parents. Due to the rapid deterioration of physical function associated with the severe pain and also evidence of SCFE, surgery was recommended. Risks and complications were thoroughly discussed. Closed reduction and internal fixation of both femoral neck fractures was carried out using 5.8-mm cannulated screws (aap Implantate AG, Berlin, Germany) as shown in Fig. 4. The surgery was performed by the leading pediatric surgeon in our institute who had limited experience in treating osteopetrotic fractures. There were no intraoperative complications. No weight bearing or hip flexion of more than 90° for a period of 5 weeks was instructed. The phase of partial to full weight bearing with crutches was then started 5 weeks postoperatively with intensive physiotherapy. Full weight bearing was reached without problems. After 8 weeks, the patient had no pain and did not limp. Daily activities were slowly resumed without problems. The mother reported that her daughter resumed playing and going to the kindergarten as before with occasional pain on the left side. Radiographs after 7 months showed a loss of fracture reposition on the left side with a coxa vara deformity and minor increased slippage of the epiphyseal plate (Fig. 5). Thus, a revision surgery was planned to review the osteosynthesis and to do a valgus osteotomy. The surgery was carried through using a locking cannulated blade plate (OrthoPediatrics, Indiana, United States) with 25° valgus. The surgery was uneventful. No weight bearing for 4 weeks was instructed. On follow-up, considerable improvement in the patient’s symptoms was reported. The patient was followed up every 3 months for the first year postoperatively. The radiological examination showed complete bony healing after 1 year (Fig. 5).

**Fig. 6:**
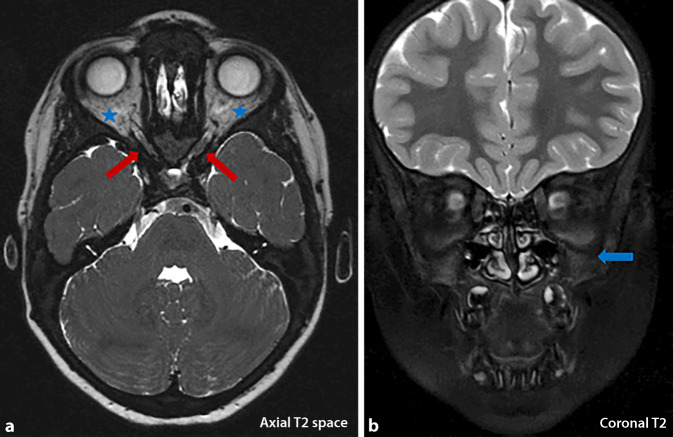
Brain MRI showing typical findings in patients with osteopetrosis. **a** On the right emphasized, pronounced narrowing of the optic canal with accompanying atrophy of the optic nerve on both sides (*blue stars*). The sheath of the optic nerve can no longer be delimited within the channel (*red arrows*). **b** Complete lack of pneumatization of the paranasal sinuses (*blue arrow*)

Regarding other manifestations of osteopetrosis, our patient suffered from diminishing vision with hyperopia and astigmatism on the right side. Magnetic resonance imaging (MRI) showed a bony narrowing of the optic canal with secondary optic nerve atrophy. As an additional finding, the patients showed a complete lack of pneumatization of the paranasal sinuses, which is also typical for patients with OP (Fig. 6). Blood tests were unremarkable, including sedimentation rate, serum urea, creatinine, electrolytes, calcium, phosphate, alkaline phosphatase, and acid phosphatase levels. Genetic counseling was offered. Since ARO was confirmed with mutations in the TCIRG1 gene, the patient was referred to our hematology division to plan treatment with HSCT. Regular visits for blood work and physical examination at the primary physician were planned.

The study was conducted according to the criteria set by the declaration of Helsinki as well as the associated university hospital where the treatment was performed. Written informed consent from the patient’s parents was acquired for the present case.

## Discussion

The present case presents successful surgical treatment in a 5-year-old patient with bilateral Salter-Harris II fractures associated with growth plate slippage secondary to confirmed OP. To the authors’ knowledge, cases with femoral neck fractures associated with SCFE secondary to OP are not reported in the current medical literature. At the time of presentation and before the genetic analysis, SCFE was initially considered as a differential diagnosis. Cooper et al. reported the first case of SCFE in 13-year-old patients with OP in 1998 treated successfully with a garden screw [24]. Hakan et al. reported a second case of bilateral SCFE in a 9-year-old boy with OP treated with cannulated screws [25]. Both of these patients suffered from ADO. Their radiographs did not show an associated fracture. Despite the Salter-Harris II fracture in our patient, we have treated her in a similar way a SCFE would have been treated in our institute using cannulated screws. This proved to be insufficient on mid-term follow-up and a more aggressive revision was necessary. Genetic testing later confirmed the diagnosis of ARO after revealing a pathogenic heterozygotic variant c.1735G > A p.(Gly579Arg) chr11.67814983 in the mother and heterozygotic variant of unclear significance c.1249G > A p.(Ala417Thr chr11:67814983) in the father. Both of these mutations in the TCIRG1 gene have been previously reported in patients with OP [14]. The clinical presentation suggests that our patient suffers from an autosomal recessive intermediate form. The disease phenotype can be rescued by providing a healthy osteoclast population via HSCT [15]. Thus, we referred the patient to the division of hematology.

Since pathological fractures due to OP usually affect the long bones, the femur is a frequently involved. Fracture patterns are transverse fractures perpendicular to the direction of stress because the dense but disorganized osteopetrotic bones cope with compression adequately but are weaker against tensile stress. Fracture healing in OP is slower due to factors such as defective callus, compromised vascularity, and altered biomechanics and remodeling. De Palma et al. histologically studied fracture healing with specimens from up to 1 year post-fracture and reported that biopsies of osteopetrotic bone showed disorganized woven bone with very low quantities of osteoclasts and osteocytes with no lamellar or Haversian systems after 1 year [27]. In the present case, even though the patient showed excellent functional results and adequate fracture osteosynthesis, full fracture healing was not reached 7 months postoperatively. Furthermore, a loss of fixation was evident on one side.

Experience of conservative therapy in treating these fractures using skin traction or a hip spica cast has been reported with a high rate of associated complications such as non-union, coxa vara and avascular necrosis of the femoral head [28, 29]. In recent years, however, two cases were reported with good results after conservative therapy. Krieg et al. reported this in a 6-year-old after 8 weeks of partial weight bearing with a follow-up of 4 years [30]. The mentioned patient had a 3-day history of pain and limping. The patient reported in our case presented with more advanced symptoms and the inability to walk anymore. It seems that in several cases, the conservative therapy is chosen by the parents after extensive discussion regarding high risk of possible complications of surgical treatment. This was also the case in a 7-year-old girl with bilateral femoral fractures reported by Kumar et al. Conservative therapy resulted in a positive outcome even though imaging showed a bilateral coxa vara deformity after 2 years [31]. The patient remained asymptomatic and thus no corrective osteotomy was performed so far. In the present case, we recommended surgery due advanced symptoms and evidence of slippage of the growth plates as well.

Although the medical literature is not conclusive, surgical treatment with closed reduction and internal fixation seems to be the preferred approach for fractures of the femoral neck in OP due to dissatisfactory early experience with conservative therapy [21, 28]. Song et al. resorted to surgical treatment using 3 Kirschner wires in a 6-year-old patient with a unilateral femoral fracture after failure of the conservative therapy with spica cast [21]. Armstrong et al. reported the treatment of 4 such fractures operatively in patients aged from 6 to 16 years with good results, whereas 3 fractures treated non-operatively developed varus and later required osteotomy [23]. Yiğit et al. reported a case series which included 4 patients with a proximal femur fracture aged 9–11 years surgically treated using either a locking compression plate or titanium elastic nail system without complications but delayed bone union with a duration of 7–12 months until complete bone healing [32]. The choice of cannulated screws in our case was made to minimize the amount of material used on the osteopetrotic bone and also to prolong the duration between the surgery and a second intervention in our pediatric patient. For the osteopetrotic bone, this was unfortunately not enough and a revision osteosynthesis with a valgus osteotomy was necessary on one side.

The dense bone structure of osteopetrotic bone is associated with a low modulus of elasticity, allowing little bony deformation. Chawla et al. reported 12 surgically treated fractures in 6 adult osteopetrotic patients. Three patients had peri-implant stress fractures, three with retained broken screws, and one case each of delayed union, non-union, and surgical site infection [20]. Ganz et al. reviewed the literature and described a list of general recommendations when operating on osteopetrotic bone in order to minimize the incidence of intraoperative complications [33]. These include taking extra care due to the brittle nature of the bones. Grasping instruments and implants like blade plates or bolt plates should be avoided. Drills and saw blades must be always kept cool. Screws should not be inserted with powered assistance. Long screws with a small diameter and titanium screws should not be preferred. Metal removal should be done only when necessary and special tools should be readily available. This should be considered in pediatric patients when the screws begin to interfere with proper growth. In adult patients, metal removal should preferably be completely avoided unless mechanical complications occur.

A limitation of the present study is reporting the experience in only one case. As a result, there was no scope for standardization. Moreover, since cases in pediatric patients are scarcely reported, a credible comparison of the surgical technique and choice of osteosynthesis equipment cannot be made.

## Conclusion

The diagnosis of OP should be considered if growth plate slippage is seen. Fractures of the femoral neck are also possible. Surgery should be the treatment of choice in fractures resulting from OP especially when functional deterioration is encountered and SCFE is present. Careful surgical planning and execution is crucial, yet delayed bone healing is still to be expected. Frequent radiological follow-up is important to rule out fixation failure and secondary bony deformity until complete bony union is reached. More reports of cases involving pediatric patients are required to form concrete guidelines for treatment.

## Abbreviations

ADO: Autosomal dominant osteopetrosis
ARO: Autosomal recessive osteopetrosis
OP: Osteopetrosis
STIR: Short tau inversion recovery

## Declarations

The mentioned study was performed in accordance with the ethical standards laid down in the 1964 Declaration of Helsinki.. For images or other information within the manuscript which identify patients, consent was obtained from them and their legal guardians.
