# Erratum zu: Pediatric patient with a bilateral Salter-Harris II fracture and slipped capital femoral epiphysis secondary to autosomal recessive osteopetrosis

**DOI:** 10.1007/s00132-022-04289-8

**Published:** 2022-08-10

**Authors:** Ayham Jaber, Martin Schwarze, Verena Steinle, Marco Götze, Sébastien Hagmann

**Affiliations:** 1 Department of Orthopedics and Trauma Surgery, Center for Orthopedics, Trauma Surgery and Spinal Cord Injury, Heidelberg University Hospital, Schlierbacher Landstr. 200a, 69118 Heidelberg, Deutschland; 2 Department of Diagnostic and interventional Radiology, Heidelberg University Hospital, Im Neuenheimer Feld 420, 69120 Heidelberg, Deutschland

**Erratum zu:**

**Orthopädie 2022**

10.1007/s00132-022-04278-x

In diesem Artikel wurden die Namen der Autoren Martin Schwarze und Marco Götze falsch angegeben. Bitte beachten Sie die korrekte Schreibweise der Autorennamen.

Der Originalbeitrag wurde korrigiert.

